# Primary Amebic Meningoencephalitis Associated with Ritual Nasal Rinsing — St. Thomas, U.S. Virgin Islands, 2012

**Published:** 2013-11-15

**Authors:** Tai Hunte, Thomas Morris, Alexandre da Silva, Azizeh Nuriddin, Govinda Visvesvara, Vincent Hill, Bonnie Mull, Lihua Xiao, Dawn Roellig, Julia Gargano, Michael Beach, Jonathan Yoder, Jennifer Cope, Jamae Morris

**Affiliations:** US Virgin Islands Dept of Health; Div of Parasitic Diseases and Malaria, Center for Global Health; Div of Foodborne, Waterborne, and Environmental Diseases, National Center for Emerging and Zoonotic Infectious Diseases; EIS Officer, CDC

On November 21, 2012, the U.S. Virgin Islands (USVI) Department of Health documented the first case and death from primary amebic meningoencephalitis (PAM) in the territory. PAM, a rare and almost universally fatal condition, results when *Naegleria fowleri*, a free-living thermophilic ameba found in warm freshwater, enters the nose and migrates to the brain. The patient was a man aged 47 years whose only reported freshwater exposures were the use of tap water for daily household activities and for ablution, a ritual cleansing that he practiced several times a day in preparation for Islamic prayer. Ablution can include nasal rinsing. On November 16, 2012, the patient had visited the emergency department with a headache; he was treated symptomatically and released. The following day, the patient returned to the emergency department by ambulance with fever, confusion, agitation, and a severe headache, for which he was admitted. Cerebrospinal fluid (CSF) studies were consistent with bacterial meningitis, and antibiotics were started. On November 18, neurologic findings included fixed nonresponsive pupils, no response in the upper or lower extremities, muted plantar responses, and no response to verbal commands. Microscopic examination of the CSF obtained from a second lumbar puncture revealed motile amebic trophozoites. CSF specimens sent to CDC for confirmatory testing were positive for *N. fowleri* by real-time polymerase chain reaction testing. On the morning of November 21, the patient was pronounced brain dead based on neurologic criteria.

During December 15–24, the USVI Department of Health and CDC conducted an environmental investigation at the patient’s home and mosque to characterize his water exposures and determine the likely source of infection. According to the patient’s roommate, the patient performed ablution, including nasal rinsing, at home and at the mosque. His household water sources were untreated groundwater from a well and untreated rainwater from a cistern; both sources were connected to the home’s plumbing system. No municipal water was piped into the home. The mosque water supply was desalinated and chlorinated municipal water. None of three samples from the mosque yielded *N. fowleri*; however, three of 17 samples from the patient’s home yielded *N. fowleri*. Water samples taken from the showerhead and the hot water heater along with the showerhead itself were positive for *N. fowleri*. None of the positive household water samples had detectable levels of free chlorine.

Detection of *N. fowleri* in the shower and hot water heater suggests that the organism had colonized the home’s plumbing system and points to the home as the likely site of exposure. Although most PAM infections are associated with recreational freshwater exposure, infection also can occur when ameba-contaminated water is introduced into the nose via nasal rinsing (1).

Ablution, including nasal rinsing, has been associated with *N. fowleri* cases globally (2). In the United States, during 2003–2012, three of 31 persons infected with *N. fowleri* became infected after performing nasal rinsing with contaminated tap water. Two of the three patients performed nasal rinsing using a neti pot or similar device (1). However, the case described in this report is the first documented U.S. case of PAM potentially associated with ablution, thus affirming the need to further understand ablution as a possible mode of *N. fowleri* transmission. Through diagnostic assistance and clinical consultation, CDC continues to support the detection of new *N. fowleri* infections and the identification of emerging modes of transmission ( http://www.cdc.gov/parasites/naegleria/cdc-at-work.html).

Measures can be taken to make water safer for ritual nasal rinsing. Using water labeled distilled or sterile, water that is boiled for 1 minute and left to cool, water filtered to remove small organisms, or water disinfected appropriately can minimize the risk for infection. Additional information regarding PAM and ablution is available at http://www.cdc.gov/parasites/naegleria/ritual-ablution.html.
